# Editorial: Clinical Applications of Anti-Mullerian Hormone and Its Measurement in Reproductive Medicine and Women’s Health

**DOI:** 10.3389/fendo.2022.879053

**Published:** 2022-03-14

**Authors:** Hang Wun Raymond Li, Scott McGill Nelson, William Leigh Ledger

**Affiliations:** ^1^Department of Obstetrics and Gynaecology, The University of Hong Kong, Queen Mary Hospital, Hong Kong, Hong Kong SAR, China; ^2^School of Medicine, University of Glasgow, Glasgow, United Kingdom; ^3^Discipline of Obstetrics & Gynaecology, School of Women’s & Children’s Health, University of New South Wales, Sydney, NSW, Australia

**Keywords:** Anti-Mullerian hormone, granulosa cells, disorders of sexual development (DSD), polycystic ovary syndrome, assisted reproduction, fertility prediction, ovarian insufficiency, fertility preservation

Anti-Mullerian hormone (AMH) is a member of the transforming growth factor-beta superfamily, which was originally identified as the factor responsible for mediating regression of the Mullerian duct in the male foetus. In the past two decades, a vast number of studies have explored its expression and roles in the adult female. It is now recognised as an important intra-ovarian regulator, being synthesised exclusively in granulosa cells of the ovarian follicles in the adult female and acting as a gate-keeper of follicular activation, growth and steroidogenesis. Its concentration in serum can now be measured using several commercially available assays and can serve as a biomarker of the functional ovarian reserve representing the follicular pool available for recruitment at any one time.

More than twenty AMH assay methods and platforms are now available commercially, with the few most commonly used platforms exhibiting good correlations between them. However, the assays frequently differ in their numerical calibration, with no international AMH standard available to harmonise the calibration, although this continues to be under active investigation. This problem of the lack of standardisation across AMH assays is a major factor limiting establishment of threshold cut-offs for various clinical applications at the moment and is reviewed by Li et al.

While serum AMH concentrations change with the age of the woman, with a general trend of decline as ovarian aging progresses, the age-specific profile of AMH also differs between ethnicities. Studies reviewed by Kotlyar and Seifer generally found higher age-specific AMH concentrations in Caucasians compared with African-American Black and Hispanic women, as well as Chinese women above the age of 25 years. Such ethnic specificity should be taken into consideration when considering clinical outcomes, particularly if ethnic specific differences in that outcome exist.

Recently, it has been recognised that AMH exists in multiple molecular isoforms, including pro-AMH, AMH_N,C_, AMH_N_ and AMH_C_. The relative abundance of the various isoforms differs between similar-sized follicles of the same woman, and even between the follicular fluid and granulosa cells within the same follicle, as reported by Mamsen et al. This poses a further potential challenge in the development of antibody-based immunoassays to detect all molecular isoforms and in establishing which are biologically active.

As for clinical utilities, the original identification of AMH as a hormone produced from the Sertoli cells of the male foetus and during early childhood identifies the utility of measuring serum AMH in conjunction with testosterone in the differential diagnosis of disorders of sexual differentiation. This aspect of AMH clinical chemistry is comprehensively reviewed and elaborated by Josso and Rey, providing a useful guide for clinical management for these conditions.

AMH, as a biomarker of the functional ovarian follicle pool, with good correlation with the sonographic antral follicle count (2-9 mm), potentially serves as a useful diagnostic tool differentiating between the various causes of anovulation, as reviewed by Capuzzo and La Marca. In particular, women with polycystic ovary syndrome (PCOS) typically have significantly higher serum AMH concentrations in line with their higher antral follicle count, while those with WHO type 3 anovulation have very low or undetectable serum AMH. Establishment of universal diagnostic cut-offs, however, is currently limited by the variability between different assay methods which remains to be solved. The role of exaggerated AMH production in the deregulation of granulosa cells, a major factor contributing to the pathogenesis of PCOS, is further elaborated by Dewailly et al.

On the other hand, the most common clinical utilisation of serum AMH measurement is for predicting ovarian response in women undergoing assisted reproduction treatment, as reviewed by Li and Nelson. Ovarian stimulation protocols can be tailored to the predicted ovarian response for individual women, although AMH has limited predictive performance for livebirth. It is suggested that AMH, used as a continuous measure in conjunction with other prognostic factors, will continue to contribute to more refined treatment algorithms.

As a biomarker of ovarian reserve, AMH has also been explored as a predictor of natural fecundability of the female. Lin et al. presented a systematic review and meta-analysis of 11 studies including 4,388 women, indicating that serum AMH concentration has poor performance in predicting natural conception.

Upon reaching the end of the reproductive span, serum AMH declines and approaches undetectable levels. While the AMH trajectory of individual women may provide some clue as to her reproductive span, the prospective prediction of timing of menopause by serum AMH in the general population is generally too imprecise to be clinically meaningful from available studies, as reviewed by de Kat et al. In the specific scenario of women undergoing cancer treatment, measuring serum AMH pre-treatment may offer some prediction of long-term ovarian function after chemotherapy, and post-treatment AMH may offer prediction of the likelihood of gonadal function recovery, as reviewed by Anderson and Su. This may impact on treatment plans, although there is yet limited data regarding preservation of fertility post-treatment.

Looking into the future from a therapeutic angle, Rodgers et al. discuss the possibility of administering AMH as a therapeutic agent for inhibiting follicular activation and hence protecting the ovarian reserve in females undergoing chemotherapy, as well as inducing regression of ovarian cancer cells that express the AMH receptor (AMHRII). These novel therapeutic targets have been tested in animal studies with some promising results, although no clinical trials in human are yet available.

These articles in this Research Topic series provides a contemporary and comprehensive collection of current knowledge and views about the clinical utilities and limitations of AMH measurement in various aspects of reproductive medicine and women’s health, as well as the potential clinical implications and applications of the AMH molecule itself. This should prompt further work to overcome the current diagnostic pitfalls and limitations, refine clinical algorithms, and establish novel therapeutic targets related to this fascinating molecule.

## Author Contributions

All authors listed have made a substantial, direct, and intellectual contribution to the work and approved it for publication.

## Conflict of Interest

The authors declare that the research was conducted in the absence of any commercial or financial relationships that could be construed as a potential conflict of interest.

## Publisher’s Note

All claims expressed in this article are solely those of the authors and do not necessarily represent those of their affiliated organizations, or those of the publisher, the editors and the reviewers. Any product that may be evaluated in this article, or claim that may be made by its manufacturer, is not guaranteed or endorsed by the publisher.

